# Role of Essential Oil-Based Mouthwashes in Controlling Gingivitis in Patients Undergoing Fixed Orthodontic Treatment. A Review of Clinical Trials

**DOI:** 10.3390/ijerph182010825

**Published:** 2021-10-15

**Authors:** Aristeidis Panagiotou, P. Emile Rossouw, Dimitrios Michelogiannakis, Fawad Javed

**Affiliations:** Department of Orthodontics and Dentofacial Orthopedics, Eastman Institute for Oral Health, University of Rochester, Rochester, NY 14620, USA; aristpanagiotou@gmail.com (A.P.); Emile_Rossouw@URMC.Rochester.edu (P.E.R.); Dimitrios_Michelogiannakis@URMC.Rochester.edu (D.M.)

**Keywords:** essential oil, mouthwash, gingivitis, oral hygiene, orthodontic appliances, fixed orthodontic treatment

## Abstract

Essential oil (EO)-based mouthwashes have been used for oral health maintenance due to their antimicrobial and anti-inflammatory properties. The aim was to review clinical trials that assessed the role of EO-based mouthwashes in controlling gingivitis in patients undergoing fixed orthodontic treatment (OT). The Patients, Interventions, Control and Outcome (PICO) format was based on the following: (a) P: Patients undergoing fixed OT (b) Intervention: EO-based mouth-wash; Control: Mouthwashes that did not contain EOs or no mouthwash (d) Outcome: Control of gingivitis measured by clinical indices. Databases were searched manually and electronically up to and including May 2021 using different medical subject subheadings. Data screening and extraction were performed. The risk of bias within randomized controlled trials was assessed using the revised Cochrane Collaboration’s risk of bias tool (RoB 2). The Risk of Bias In Non-randomized Studies—of Interventions (ROBINS-I) tool was used for non-randomized controlled trials. Disagreements related to literature search and RoB evaluations were resolved via discussion. Six clinical studies were included. Four studies showed that Listerine^®^ is effective in controlling gingivitis in patients undergoing fixed OT. One study reported that the use of 5% Fructus mume mouthwash resulted in a significant reduction in gingival bleeding. Two mouthwashes that contained 1% *Matricaria chamomilla* L. and 0.5% *Zingiber officinale* were also found to be efficient in controlling gingival bleeding. Four, one and one studies had a low, moderate and high RoB, respectively. In conclusion, EO-based mouthwashes seem to be effective for the management of gingivitis among patients undergoing fixed OT. Further well-designed and power-adjusted clinical trials are needed.

## 1. Introduction

Essential oils (EOs) are organic compounds that are extracted from plants with various distillation methods [1]. Historically, they have been utilized in process of manufacturing perfumes due to their strong and characteristic scent, as well as in food and beverage industries [2]. The EO-derivatives possess anti-inflammatory and antimicrobial properties and have been used in the field of clinical dentistry and related research [3]. Lavender, peppermint, cinnamon and clove oils have been shown to have an inhibitory effect on different bacteria and fungi [3]. Recently, EO-derivatives were found to be efficient in the management of orofacial pain due to their analgesic properties [4,5]. Furthermore, studies indicated that EOs are capable of the management of dental anxiety before certain surgical procedures [6,7].

Patients undergoing fixed orthodontic treatment (OT) are more prone to gingival inflammation because fixed orthodontic appliances are bulky and create a favorable environment for plaque accumulation [8,9]. Mechanical plaque removal poses a challenge for orthodontic patients and different strategies have been implemented in order to control plaque formation, prevent the development of gingivitis and maintain oral health [9]. More specifically, chemotherapeutic agents with antimicrobial properties, such as 0.12% chlorhexidine (CHX), have been proposed as an adjunct to the standard oral hygiene protocol [10]. However, prolonged use of these agents has been associated with side effects, such as hypersensitivity reactions, burning sensation and changes in taste and tooth color [11,12]. Another potential approach for the management of oral health in orthodontic patients is the use of EO-containing mouthwashes due to their antimicrobial and anti-inflammatory properties [13]. A recent study [13] investigated the effectiveness of mouthwash with 1% Matricaria chamomilla L. (MTC) in the management of gingivitis during OT, by comparing it to CHX and a placebo mouthwash. The authors concluded that the use of both CHX and MTC mouthwash significantly reduced gingival inflammation compared to the use of the placebo mouthwash [13]. Listerine^®^ is another EO-containing mouthwash that has been studied in the literature [14]. In the study by Tufekci et al. [10], it was found that orthodontic patients who used Listerine^®^ along with tooth brushing and flossing exhibited significantly better oral hygiene compared to those that did not use any mouthwash. Based on these studies [10,13], it is speculated that there is a potential role of EO-based mouthwashes in controlling gingivitis during fixed OT. A vigilant review of the pertinent indexed literature revealed that there are no studies that have reviewed the effectiveness of EO-containing mouthwashes in the management of gingivitis among orthodontic patients.

With this background, the aim of the present study was to review studies that assessed the role of EO-based mouthwashes in controlling gingivitis in patients undergoing fixed OT.

## 2. Materials and Methods

In the present study, a review of pertinent indexed literature was performed. Therefore, the study protocol was exempted from prior approval from an institutional review board. The study protocol was registered in PROSPERO (registration number: CRD42021252935).

The addressed focused question was “Are EO-based mouthwashes effective in controlling gingivitis in patients undergoing fixed OT?”.

The Patients, Interventions, Control and Outcome (PICO) format was based on the following: (a) P: Patients undergoing fixed OT (b) Intervention: EO-based mouthwash; Control: Mouthwashes that did not contain EOs (CHX, povidone-iodine, placebo) or no mouthwash (d) Outcome: Control of gingivitis measured by clinical indices (Plaque Index [10,11], Visible Plaque Index [13,15], Modified Plaque Index [16], Bleeding Index [10,11], Gingival Bleeding Index [13,17], Gingival Index [15], and Modified Gingival Index [10,11,16].

The inclusion criteria were as follows: (a) randomized clinical trials (RCTs), (b) non-randomized controlled trials (non-RCTs), (c) patients undergoing fixed OT, (d) use of EO-based mouthwashes (test group) and (e) use of mouthwashes that did not contain EOs (CHX, povidone-iodine, placebo) or no mouthwash (control group). Letters to the Editor, commentaries, case reports/series, reviews and studies published in non-indexed databases were excluded.

The literature search was carried out using the PRISMA guidelines [18]. The PubMed/Medline databases were searched by two investigators (AP and FJ) without time and language barriers up to and including May 2021. Different combinations of the following keywords were used during the literature search: (a) Essential oil; (b) essential oils; (c) peppermint; (d) clove; (e) lavender; (f) chamomile; (g) cinnamon; (h) melaleuca; (i) thyme; (j) eucalyptus; (k) lemon; (l) fixed orthodontic treatment; (m) orthodontics; (n) oral hygiene; (o) gingivitis. These keywords were combined using Boolean operators (OR, AND) to expand the search results. Two investigators (AP and FJ) independently screened the titles and abstracts of studies identified, and independently read full texts of relevant studies. Reference lists of potentially relevant original studies were hand-searched to identify studies that could have been missed during the initial search. Disagreements related to the inclusion of studies were resolved via discussion and consultation with a third researcher (DM).

Data extraction was independently performed by 2 authors (AP and FJ). The pertinent information was charted as follows: (a) reference; (b) type of study; (c) number of participants; (d) subject characteristics (mean age and gender); (e) clinical indices; (f) duration of follow-up; (g) study groups based upon the type of mouthwash used; (h) power analysis; (i) type, concentration and daily frequency of mouthwash usage; (j) main outcomes and conclusions; and (k) risk of bias assessment. Any disagreements related to the data extraction process were again resolved through discussion among the authors (AP and FJ) and consultation with a third author (DM).

Two investigators (AP and FJ) used the revised Cochrane Collaboration’s risk of bias tool (RoB 2) [19] in order to determine the risk of bias within RCTs. The ROBINS-I tool (Risk of Bias In Non-randomized Studies—of Interventions) was used for non-RCTs. Disagreements were again resolved via discussion with a third researcher (DM).

## 3. Results

### 3.1. Study Selection

**Figure 1:**
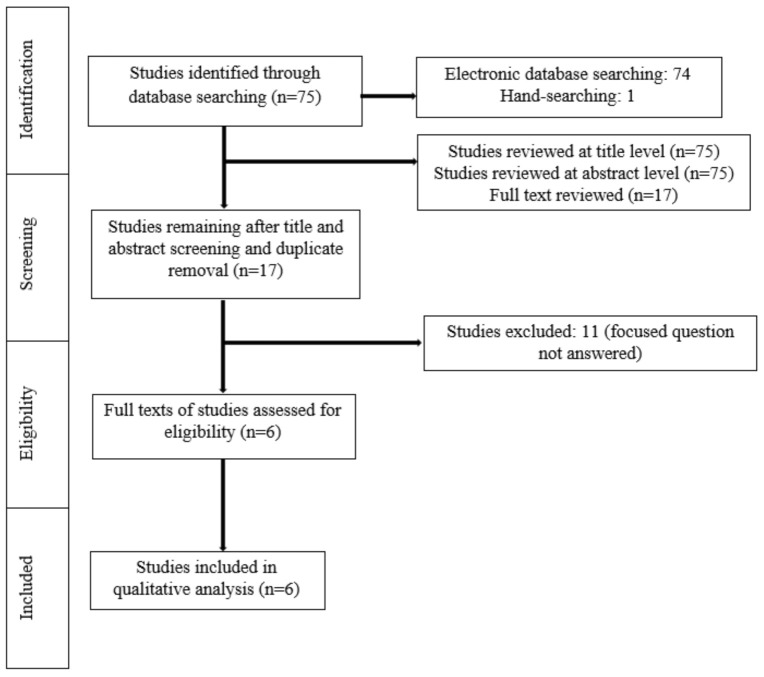
Study selection according to PRISMA guidelines.

A meticulous search of indexed literature revealed 63 studies. After title and abstract screening and removal of the duplicate studies, 17 studies were retrieved and evaluated in more detail. Eleven studies were excluded after full-text evaluation because they did not meet the eligibility criteria. In total, six studies (three RCTs, three non-RCTs) were included and processed for data extraction (Figure 1).

### 3.2. General Characteristics of the Clinical Trials Assessed

Random allocation of the participants to the groups was performed in three studies [11,13,15]. In the study by Bauer Faria et al. [17], 31 patients undergoing fixed OT used 3 different mouthwashes in a random order. Initially, all the participants were instructed to use a mouthwash containing 0.5% *Zingiber officinale* (ZO), then 0.12% CHX and finally a placebo mouthwash [17]. Tufekci et al. (10), reported that the age and gender of the participants could play a significant role in their compliance and could therefore influence the findings of the study. As a result, patients were matched for both these variables [10]. In the study by Akbulut [16], no randomization of the participants was mentioned. In five studies [10,11,13,15,17], the number of participants ranged between 30 and 79. In the study by Akbulut [16], the groups were formed according to the number of mini screws. More specifically, 38 patients were divided into 4 groups, with each group consisting of 15 mini screws [16]. The number of males and females ranged between 4 and 20 and 17 and 27, respectively [10,13,15,16,17]. One study [11] did not report the number of male and female participants. In five studies [10,11,13,15,17], the age of the patients ranged between 10 and 64 years. In the study by Akbulut [16], the age of the participants was not mentioned. In all studies [10,11,13,15,16,17], a variation was identified at the clinical indices used to assess the gingival status of the participants (Plaque Index, Visible Plaque Index, Modified Plaque Index, Bleeding Index, Gingival Bleeding Index, Gingival Index, and Modified Gingival Index). The duration of follow-up also varied among studies [10,11,13,15,16,17] from baseline (0 days of treatment) to 180 days of intervention (Table 1). All studies [10,11,13,15,16,17] included participants who used a mouthwash that contained EOs (test group) and those who either used mouthwashes that did not contain EOs or did not use any mouthwash (control group) during OT with fixed appliances. A power analysis was performed in three studies [10,11,17] (Table 2).

### 3.3. Type, Concentration and Daily Frequency of Mouthwash Usage

The participants in the test group used a variation of mouthwashes that contained EOs, including Listerine^®^ [10,11,15,16], 1% MTC [13], 0.5% ZO [17] and 2.5% Fructus mume (FM) [11]. Moreover, different mouthwashes were used by subjects in the control group, including 0.12% CHX [13,16,17], 7.5% povidone-iodine [16] and placebo [13,15,17]. In four studies [10,11,15,16], the participants in the control group were instructed to brush and floss, but not use any mouthwash. In four studies [10,11,13,15] the mouthwashes were used twice daily. However, two studies did not report a specific protocol regarding the daily frequency of usage of the mouthwashes [16,17] (Table 3).

### 3.4. Main Study Outcomes

In the study by Tufekci et al. [10], all clinical indices were significantly higher in the group that did not use a mouthwash compared with the Listerine^®^ group after 90 and 180 days. It was concluded that Listerine^®^ is efficient in controlling plaque accumulation and gingivitis in patients undergoing fixed OT [10]. In the study by Alves et al. [15], Listerine^®^ was also found to be more successful in controlling gingivitis compared with a placebo mouthwash. Chen et al. [11] reported that the use of both Listerine^®^ and FM mouthwashes resulted in a significant reduction in bleeding of gingival tissue compared with the use of no mouthwash. Furthermore, Listerine^®^ and FM mouthwashes were found to be equally efficient in promoting oral health [11]. In another study [16], the use of both Listerine^®^ and CHX significantly improved the oral hygiene status of patients with orthodontic mini screws compared with the use of povidone iodine or no mouthwash. Goes et al. [13] reported that both CHX and MTC mouthwashes were more effective in controlling plaque accumulation and gingival bleeding compared with a placebo mouthwash. Moreover, there were no differences when CHX was compared with the MTC mouthwash [13]. In the study by Bauer Faria et al. [17], ZO mouthwash had higher efficiency in controlling gingival inflammation compared with CHX and a placebo mouthwash (Table 4).

### 3.5. Risk of Bias

**Figure 2:**
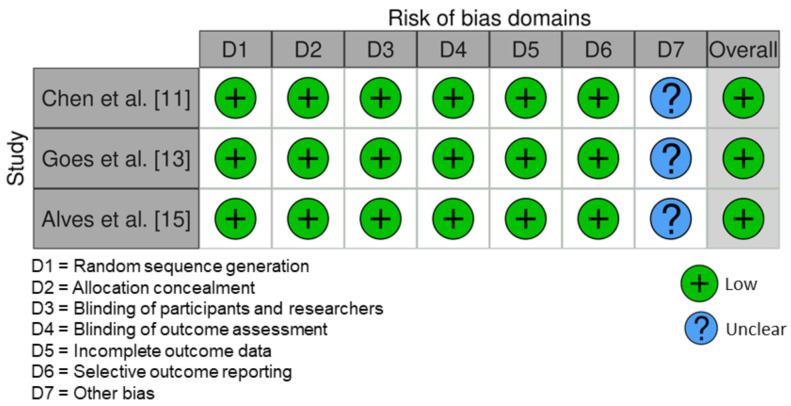
Risk of bias of the included randomized controlled trials.

**Figure 3:**
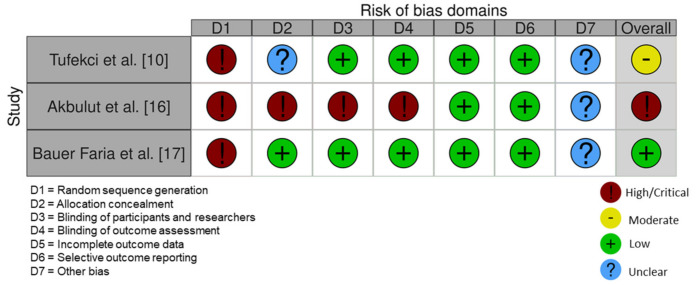
Risk of bias of the included non-randomized controlled trials.

All RCT studies had a low risk of bias [11,13,15] (Figure 2). One non-RCT [17] had a low risk of bias according to ROBINS-I tool. The studies by Tufekci et al. [10] and Akbulut [16] had a moderate and high risk of bias, respectively (Figure 3).

## 4. Discussion

Oral health maintenance can be problematic in orthodontic patients due to the increased plaque retention around the orthodontic appliances [8,9]. Based on the studies of the present review, it can be assumed that EO-based mouthwashes are efficient in controlling gingivitis during fixed OT and can be used as an adjunct to the regular oral hygiene protocol in these patients. Approximately 66% of the studies [10,11,15,16] evaluated the effect of Listerine^®^ in oral health maintenance in orthodontic patients. Tufekci et al. [10] reported that Listerine^®^ can be advantageous because all clinical indices (BI, MGI and PI) in the Listerine^®^ group had a significantly lower value compared with the group that did not use a mouthwash after 3 and 6 months. This comes into agreement with the study by Chen et al. [11], which used the same methodology as the previous study. However, in the study by Chen et al. [11], there were no significant differences in MGI and PI between the two groups mentioned before. In the study by Alves et al. [15], Listerine^®^ was also found to be beneficial in reducing plaque accumulation and inflammation of the gingival tissue compared with a placebo mouthwash and the use of no mouthwash. The same conclusion was reached in the study by Akbulut [16] in orthodontic patients with mini-screws. Moreover, there was no significant difference when Listerine^®^ was compared to CHX and povidone-iodine mouthwash [16]. There were two more studies [13,17] that compared EO- based mouthwashes to CHX. In the study by Goes et al. [13], it was found that MTC mouthwash had comparable anti-inflammatory efficacy with CHX. On the contrary, Bauer Faria et al. [17] reported that ZO mouthwash had a higher one compared with CHX. According to Chen et al. [11], FM mouthwash was equally efficient with Listerine^®^ in the management of gingivitis.

However, these results should be evaluated carefully due to the variability in the methodology of the studies [10,11,13,15,16,17]. Firstly, there was a great variation in the mouthwashes used by the participants in the control groups among the studies [10,11,13,15,16]. Half of the studies [13,16,17] compared EO-based mouthwashes with CHX, while in four of them the participants in the control group did not use a mouthwash [10,11,15,16]. Moreover, a placebo mouthwash was used in three studies [13,15,17]. The studies [10,11,13,15,16,17] also varied widely in the duration of follow-up. In the study by Bauer Faria et al. [17], the participants used each mouthwash for a period of 7 days. However, in two studies [10,11], the mouthwashes were used for a considerably longer time of 180 days. Furthermore, Tufekci et al. [10] and Chen et al. [11] used the same clinical indices in order to assess the effect of each mouthwash. However, in the remaining studies [13,15,16,17] a wide variety of clinical indices were implemented according to the authors’ preference. In this regard, the results should be interpreted with caution and further well-designed RCTs are needed. Prolonged use of CHX, which is considered to be the gold standard mouthwash, has been associated with certain side effects, such as burning sensation and changes in taste and tooth color [12]. This comes into agreement with the study by Goes et al. [13] in which five patients, that used CHX, reported burning or taste alterations after 14 days. However, no side effect was mentioned by the subjects that used the MTC mouthwash [13]. On the other hand, Bauer Faria et al. [17] reported that both the participants that used CHX and ZO mouthwashes had low taste tolerance. In the rest of the studies [10,11,15,16], no adverse reactions were mentioned by the authors.

A major limitation of the present review is that only six studies [10,11,13,15,16,17] fulfilled the eligibility criteria and were processed for data extraction. Of these, 50% were non-RCTs [10,16,17]. From the authors’ perspective, this limitation may have compromised the quality of available evidence. It is also noteworthy that there was heterogeneity in several parameters, such as the type of mouthwash, concentration of mouthwash and daily frequency of usage amongst the studies assessed [10,11,13,15,16,17]. In this context, a meta-analysis of the qualitative data could not be performed. Power analysis is a fundamental tool that estimates the appropriate number of subjects that are needed in order to detect statistically significant differences [20]. A careful evaluation of the studies, revealed that a power analysis was not performed in 50% of the studies [10,11,17]. As a result, the statistically significant differences reported in these studies should be addressed very carefully. Furthermore, blinding of both the examiners and participants, which is an integral part of a well-designed study, was performed in two studies [13,15]. In the studies by Tufekci et al. [10] and Chen et al. [11], only the examiners were blinded; whereas in the remaining studies [16,17], neither the examiners nor the participants were blinded. Therefore, the outcomes should be cautiously interpreted due to the potentially increased risk of bias.

## 5. Conclusions

Based upon the limited evidence available, EO-based mouthwashes seem to be effective for the management of gingivitis among patients undergoing fixed OT. Further well-designed and power-adjusted clinical trials are needed.

## Figures and Tables

**Table 1:** General characteristics of the clinical trials assessed.

Authors	Type of Study	Participants	Gender	Age in Years (Range)	Clinical Indices	Duration of Follow-Up
Tufekci et al. (2008) [10]	Non-RCT	47	20 M 27 F	16.6 years * (10–64)	PI, BI and MGI	90 and 180 days
Chen et al. (2013) [11]	RCT	79	NR	17.7 ± 3.9 years	PI, BI and MGI	90 and 180 days
Goes et al. (2016) [13]	RCT	30	4 M 26 F	28.8 ± 3.28 years (10–40)	VPI and GBI	15 days
Alves et al. (2010) [15]	RCT	30	10 M 20 F	12–21 years	VPI and GI	60 days
Akbulut ** (2020) [16]	Non-RCT	38 (60 mini screws)	18 M 20 F	NR	MPI and MGI	21 days
Bauer Faria et al. (2021) [17]	Non-RCT	31	14 M 17 F	19.96 years (12–35)	GBI	7 days

* Refers to the age of the initial sample; ** There were 15 mini-screw implants per group. Number of patients was not reported; NR, Not reported; RCT, Randomized controlled trial; Non-RCT, Non-randomized controlled trial; M, Males; F, Females; PI, Plaque Index; VPI, Visible Plaque Index; MPI, Modified Plaque Index; BI, Bleeding Index; GBI, Gingival Bleeding Index; GI, Gingival Index; MGI, Modified Gingival Index.

**Table 2:** Test and control groups and power analysis.

Authors (Year)	Test Group (*n* = Number of Patients)	Control Group (*n* = Number of Patients)	Power Analysis
Tufekci et al. (2008) [10]	EO-based MW (*n* = 24)	No MW (*n* = 23)	Yes
Chen et al. (2013) [11]	EO-based MW 1 (*n* = 28) EO-based MW 2 (*n* = 25)	No MW (*n* = 26)	Yes
Goes et al. (2016) [13]	EO-based MW (*n* = 10)	CHX (*n* = 10) Placebo MW (*n* = 10)	No
Alves et al. (2010) [15]	EO-based MW (*n* = 10)	Placebo MW (*n* = 10) No MW (*n* = 10)	No
Akbulut (2020) [16]	EO-based MW (*n* = NR)	CHX (*n* = NR) Povidone-iodine MW (*n* = NR) No MW (*n* = NR)	No
Bauer Faria et al. (2021) [17]	EO-based MW (*n* = 31)	CHX (*n* = 31) Placebo MW (*n* = 31)	Yes

EO, Essential oil; MW, Mouthwash; CHX, Chlorhexidine; NR, Not reported.

**Table 3:** Type, concentration and daily frequency of mouthwash usage.

Authors (Year)	Type of MW		Concentration of MW		Daily Frequency of Usage	
	Test-Group	Control-Group	Test-Group	Control-Group	Test-Group	Control-Group
Tufekci et al. (2008) [10]	Listerine^®^	No MW	NR	NA	2× daily	NA
Chen et al. (2013) [11]	Listerine^®^ Fructus mume	No MW	NR 2.5%	NA	2× daily 2× daily	NA
Goes et al. (2016) [13]	Matricaria chamomilla L.	CHX Placebo	1%	0.12% NR	2× daily	2× daily 2× daily
Alves et al. (2010) [15]	Listerine^®^	Placebo No MW	NR	NR NA	2× daily	2× daily NA
Akbulut (2020) [16]	Listerine^®^	CHX Povidone iodine No MW	NR	0.12% 7.5% NA	NR	NR NR NA
Bauer Faria et al. (2021) [17]	*Zingiber officinale*	CHX Distilled water	0.5%	0.12% NA	NR	NR NA

MW, Mouthwash; NR, Not reported; NA, Not applicable; CHX, Chlorhexidine.

**Table 4:** Main study outcomes.

Authors (Year)	Main Outcomes	Side-Effects/Complications	Conclusion
Tufekci et al. (2008) [10]	BI, MGI and PI were significantly higher in the group that did not use a mouthwash compared with the Listerine^®^ group after 90 and 180 days.	N/R	Listerine^®^ is effective in decreasing plaque accumulation and gingival bleeding in orthodontic patients.
Chen et al. (2013) [11]	BI was significantly lower in Listerine^®^ and FM groups compared with the group that did not use a mouthwash after 180 days. MGI significantly decreased in both FM and Listerine^®^ groups after 90 days. There was no significant difference in PI, BI and MGI in patients that used Listerine^®^ and FM	No complications were mentioned by the patients.	Listerine^®^ and FM mouthwashes lead to decreased bleeding of gingival tissue in patients undergoing fixed orthodontic treatment.
Goes et al. (2016) [13]	GBI and VPI were significantly higher in the placebo group compared with the MTC and CHX groups. No significant difference in GBI and VPI in patients that used MTC and CHX.	In the CHX group, five patients reported burning or alterations in taste after 14 days. In the placebo group, one patient reported tongue numbness on day 1.	MTC and CHX are effective in reducing gingival bleeding and oral biofilm accumulation in patients with gingivitis. Anti-inflammatory efficacy of MTC and CHX are comparable.
Alves et al. (2010) [15]	GI significantly decreased in the Listerine^®^ group compared with the placebo mouthwash group. GI and VPI significantly decreased in the Listerine^®^ group compared with the group that did not use a mouthwash.	N/R	Listerine^®^ is an effective adjunct to oral hygiene in patients undergoing orthodontic treatment.
Akbulut (2020) [16]	MPI and MGI significantly decreased in CHX and Listerine^®^ groups after 3 weeks. MPI and MGI were significantly lower in CHX, Listerine^®^ and povidone-iodine groups compared with the group that did not use a mouthwash.	N/R	CHX, Listerine^®^ and povidone-iodine are effective in promoting oral health in patients with orthodontic mini screws.
Bauer Faria et al. (2021) [17]	GBI was significantly higher in the CHX group compared with the ZO group after 7 days.	ZO and CHX have a low taste tolerance.	ZO reduces gingival bleeding and oral biofilm accumulation.

CHX: Chlorhexidine; MTC: *Matricaria chamomilla* L; FM: Fructus mume; ZO: *Zingiber officinale*; PI: Plaque index; VPI: Visible plaque index; MPI: Modified plaque index; GI: Gingival index; MGI: Modified gingival index; GBI: Gingival bleeding index; BI: Bleeding index; NR: Not reported.

## Data Availability

Data is available at reasonable request.

## References

[1] Wińska K., Mączka W., Łyczko J., Grabarczyk M., Czubaszek A., Szumny A. (2019). Essential Oils as Antimicrobial Agents—Myth or Real Alternative?. Molecules.

[2] Elshafie H.S., Camele I. (2017). An Overview of the Biological Effects of Some Mediterranean Essential Oils on Human Health. BioMed Res. Int..

[3] Dagli N., Dagli R.J., Mahmoud R.S., Baroudi K. (2015). Essential oils, their therapeutic properties, and implication in dentistry: A review. J. Int. Soc. Prev. Community Dent..

[4] Quintans J.S., Brito R.G., Aquino P.G., França P.H., Siqueira-Lima P.S., Santana A.E., Ribeiro E.A., Salvador M.J., Araújo-Júnior J.X., Quintans-Júnior L.J. (2014). Antinociceptive activity of Syzygium cumini leaves ethanol extract on orofacial nociception protocols in rodents. Pharm. Biol..

[5] Bonjardim L.R., Silva A.M., Oliveira M.G.B., Guimarães A.G., Antoniolli A.R., Santana M.F., Serafini M.R., Santos R.C., Araújo A.A.S., Estevam C.S. (2011). Sida cordifolia Leaf Extract Reduces the Orofacial Nociceptive Response in Mice. Phytotherapy Res..

[6] Zabirunnisa M., Gadagi J.S., Gadde P., Koneru J., Myla N., Thatimatla C. (2014). Dental patient anxiety: Possible deal with Lavender fragrance. J. Res. Pharm. Pract..

[7] Hasheminia D., Motamedi M.R.K., Ahmadabadi F.K., Hashemzehi H., Haghighat A. (2014). Can Ambient Orange Fragrance Reduce Patient Anxiety During Surgical Removal of Impacted Mandibular Third Molars?. J. Oral Maxillofac. Surg..

[8] Martin B.J., Campbell P.M., Rees T.D., Buschang P.H. (2016). A randomized controlled trial evaluating antioxidant–essential oil gel as a treatment for gingivitis in orthodontic patients. Angle Orthod..

[9] Santamaria M., Petermann K.D., Vedovello S.A.S., Degan V., Lucato A., Franzini C.M. (2014). Antimicrobial effect of Melaleuca alternifolia dental gel in orthodontic patients. Am. J. Orthod. Dentofac. Orthop..

[10] Tufekci E., Casagrande Z.A., Lindauer S.J., Fowler C.E., Williams K.T. (2008). Effectiveness of an Essential Oil Mouthrinse in Improving Oral Health in Orthodontic Patients. Angle Orthod..

[11] Chen Y., Wong R.W.K., Seneviratne C.J., Hagg U., McGrath C.P.J., Samaranayake L.P. (2012). The effects of natural compounds-containing mouthrinses on patients with fixed orthodontic appliance treatment: Clinical and microbiological outcomes. Int. J. Paediatr. Dent..

[12] Kotsailidi E.A., Kalogirou E.-M., Michelogiannakis D., Vlachodimitropoulos D., Tosios K.I. (2020). Hypersensitivity reaction of the gingiva to chlorhexidine: Case report and literature review. Oral Surg. Oral Med. Oral Pathol. Oral Radiol..

[13] Goes P., Dutra C.S., Lisboa M.R.P., Gondim D.V., Leitão R., Brito G.A.C., Rego R.O. (2016). Clinical efficacy of a 1% Matricaria chamomile L. mouthwash and 0.12% chlorhexidine for gingivitis control in patients undergoing orthodontic treatment with fixed appliances. J. Oral Sci..

[14] Vlachojannis C., Chrubasik-Hausmann S., Hellwig E., Al-Ahmad A. (2015). A Preliminary Investigation on the Antimicrobial Activity of Listerine^®^, Its Components, and of Mixtures Thereof. Phytother. Res..

[15] Zenóbio E., Alves K.M., Goursand D., Cruz R.A. (2010). Effectiveness of Procedures for the Chemical-Mechanical Control of Dental Biofilm in Orthodontic Patients. J. Contemp. Dent. Pract..

[16] Akbulut Y. (2020). The effects of different antiseptic mouthwash on microbiota around orthodontic mini-screw. Niger. J. Clin. Pract..

[17] Bauer Faria T.R., Furletti-Goes V.F., Franzini C.M., de Aro A.A., de Andrade T.A.M., Sartoratto A., de Menezes C.C. (2021). Anti-inflammatory and antimicrobial effects of *Zingiber officinale* mouthwash on patients with fixed orthodontic appliances. Am. J. Orthod. Dentofac. Orthop..

[18] Page M.J., McKenzie J.E., Bossuyt P.M., Boutron I., Hoffmann T.C., Mulrow C.D., Shamseer L., Tetzlaff J.M., Akl E.A., Brennan S.E. (2021). The PRISMA 2020 statement: An updated guideline for reporting systematic reviews. BMJ.

[19] Sterne J.A.C., Savović J., Page M.J., Elbers R.G., Blencowe N.S., Boutron I., Cates C.J., Cheng H.-Y., Corbett M.S., Eldridge S.M. (2019). RoB 2: A revised tool for assessing risk of bias in randomised trials. BMJ.

[20] Beck T.W. (2013). The Importance of A Priori Sample Size Estimation in Strength and Conditioning Research. J. Strength Cond. Res..

